# Niche-driven evolution of metabolic and life-history strategies in natural and domesticated populations of *Saccharomyces cerevisiae*

**DOI:** 10.1186/1471-2148-9-296

**Published:** 2009-12-22

**Authors:** Aymé Spor, Thibault Nidelet, Jonattan Simon, Aurélie Bourgais, Dominique de Vienne, Delphine Sicard

**Affiliations:** 1 Univ Paris-Sud, UMR de Génétique Végétale, INRA/Univ Paris-Sud/CNRS/AgroParisTech, Ferme du Moulon, Gif-sur-Yvette, F-91190, France

## Abstract

**Background:**

Variation of resource supply is one of the key factors that drive the evolution of life-history strategies, and hence the interactions between individuals. In the yeast *Saccharomyces cerevisiae*, two life-history strategies related to different resource utilization have been previously described in strains from different industrial origins. In this work, we analyzed metabolic traits and life-history strategies in a broader collection of yeast strains sampled in various ecological niches (forest, human body, fruits, laboratory and industrial environments).

**Results:**

By analysing the genetic and plastic variation of six life-history and three metabolic traits, we showed that *S. cerevisiae *populations harbour different strategies depending on their ecological niches. On one hand, the forest and laboratory strains, referred to as extreme "ants", reproduce quickly, reach a large carrying capacity and a small cell size in fermentation, but have a low reproduction rate in respiration. On the other hand, the industrial strains, referred to as extreme "grasshoppers", reproduce slowly, reach a small carrying capacity but have a big cell size in fermentation and a high reproduction rate in respiration. "Grasshoppers" have usually higher glucose consumption rate than "ants", while they produce lower quantities of ethanol, suggesting that they store cell resources rather than secreting secondary products to cross-feed or poison competitors. The clinical and fruit strains are intermediate between these two groups.

**Conclusions:**

Altogether, these results are consistent with a niche-driven evolution of *S. cerevisiae*, with phenotypic convergence of populations living in similar habitat. They also revealed that competition between strains having contrasted life-history strategies ("ants" and "grasshoppers") seems to occur at low frequency or be unstable since opposite life-history strategies appeared to be maintained in distinct ecological niches.

## Background

Resource supply is one of the most important ecological factors that drive the evolution of organisms. Therefore the metabolic pathways implicated in external resource consumption are expected to have an essential role in the evolution of life-history traits. Two different strategies for exploiting resources are commonly described. In the "selfish" strategy the individuals are consuming quickly resources and increase their own reproduction rate. In the "cooperative" strategy the individuals save resources for the whole group at the expense of their own reproduction rate. In the latter case, individuals are supposed to exploit resource slowly but efficiently. When individuals share common resources, there is a dilemma in which individuals that act selfishly consume rapidly the common resources, reducing the fitness of the whole group, which leads to what has been called the "tragedy of the commons" [1]. The central principle of such a "social conflict" is that there must be a trade-off between individual short-term and population long-term interests. This metaphor reflecting a dilemma between individual and population interests has been used in a vaste diversity of fields in science, from the evolution of competition and cooperation in insect societies [2] to intra-genomic conflict [3], and even to propose a solution to alleviate and improve the work of reviewers [4].

In the case of heterotrophic microorganisms, the existence of a dilemma between selfish and cooperative strategies has been proposed to result from a metabolic trade-off between the rate and efficiency of resource use [5,6]. A high rate of ATP production per unit of time is associated with a high reproduction rate and is considered as a selfish strategy, whereas a high yield of ATP production, *i.e*. the number of units of ATP per unit of resource consumed, is associated with a low reproduction rate but with a high biomass production, and is considered a cooperative strategy. The evolutionary significance of such a trade-off has been studied using both experimental and theoretical approaches in the context of evolutionary game theory [7]. The outcome of competition between selfish and cooperative strains for a common resource is that a selfish strain with a high rate of ATP production always wins over the prudent strain with a high yield of ATP production [8-10]. The maintenance of highly efficient cooperative strains is only possible in case of spatially structured environment or in case of density-dependent cost of selfish metabolism (toxins productions for instance) [9-11].

The yeast *Saccharomyces cerevisiae *is able to use two main metabolic strategies. The respiration allows a high efficiency of resource conversion into energy but at low rate. The fermentation is associated with a low efficiency of resource conversion into energy but a high metabolic rate. Mathematical modelling as well as construction of engineered strains of *S. cerevisiae*, either respirer or respiro-fermenter, have shown that coexistence or competitive exclusion between these two strategies could occur [11]. The analysis of these two metabolic strategies in natural populations had not yet been carried out. Although the genomic structure of *S. cerevisiae *populations begins to be studied [12-17], very few studies are dedicated to the phenotypic characterisation of yeast populations coming from different ecological niches. We showed in a previous paper that the genetic variability of metabolic and life-history traits distributes the industrial strains between two extreme life-history strategies [18]. The "grasshopper" strains have a high specific glucose consumption rate in fermentation, a large cell size but a low carrying capacity, while the "ant" strains have a low specific glucose consumption rate, a small cell size and a high carrying capacity. In other words, the "grasshoppers" behave as selfish strains allocating resource to their own cell size, while the "ants" behave as cooperative strains saving resource allowing a high population size to be reached.

The presence of these strategies in other habitats than industrial ones has not yet been addressed. Moreover, because of lack of genetic variation on reproduction rate in the studied collection of industrial strains, the previous data did not allow to ask question about the relationship between metabolic strategies and reproduction rate. Finally, life-history traits were studied in fermentation and correlated variation for performances during respiration was not studied.

In this paper, we asked whether different metabolic and life-history strategies could be found in the broad range of habitats of *S. cerevisiae*. We analyzed the plastic and genetic components of six life-history traits (intrinsic reproduction rate, carrying capacity, cell size, reproduction rate in fermentation, time of the shift between fermentation and respiration, and reproduction rate in respiration) and three metabolic traits (glucose consumption rate, ethanol production, fermentation yield) in 19 strains coming from forest, human body, laboratory, fruits and various industrial origins [18]. Strains were grown in two culture media differing in their amount of resource (1% and 15% glucose) allowing both fermentation and respiro-fermentation processes to be investigated.

## Methods

### Biological Material

Nineteen diploid strains of *S. cerevisiae *were chosen from the CIRM-Levures (Centre International de Ressources Microbiennes, Thiverval-Grignon, France), the *Saccharomyces *Genome Resequencing Project collection (Sanger Institute, Cambridge, UK) and Justin Fay's Collection (Washington University in St. Louis - School of Medicine, St. Louis, USA). The collection included strains isolated from forest, coconut and cactus fruits, human body, laboratory, brewery and winery, and has been formed in order to group together at least 3 strains from each type of habitat (Table 1). For each strain, a reference stock was conserved at -80°C in our lab.

**Table 1:** Collection of strains of *Saccharomyces *cerevisiae

Strains ID	Geographical Origin	Habitat	Description	Collection
227	Netherlands	Industrial	Brewery, Top	CIRM-Levures^1^
157	Spain	Industrial	Vinery (Grapes)	CIRM-Levures^1^
NCYC110	West Africa	Industrial	Ginger Beer from *Z officinale*	SGRP^2^
YJM320	USA	Clinical Isolate	unknown	Fay Collection^3^
YJM421	USA	Clinical Isolate	Ascitic Fluids	Fay Collection^3^
322134S	Newcastle, UK	Clinical Isolate	Thorat-Sputum	SGRP^2^
YJM981	Bergamo, Italy	Clinical Isolate	Vaginale	SGRP^2^
IL-01	Illinois, USA	Forest	Soil	Fay Collection^3^
YPS128	Pennsylvania, USA	Forest	Oak Exudate	SGRP^2^
YPS606	Pennsylvania, USA	Forest	Oak Exudate	SGRP^2^
NC-02	North Carolina, USA	Forest	Oak Exudate	Fay Collection^3^
T7	Missouri, USA	Forest	Oak Exudate	Fay Collection^3^
YPS1009	New Jersey, USA	Forest	Oak Exudate	Fay Collection^3^
Y10	Philippines	Fruit	Coconuts	Fay Collection^3^
UWOPS83-2421	Hawaii	Fruit	*Opuntia megacantha*	SGRP^2^
UWOPS83-787.3	Bahamas	Fruit	*Opuntia stricta*	SGRP^2^
S288C	California, USA	Laboratory	Rotting fig	SGRP^2^
Y55	France	Laboratory	Wine	SGRP^2^
SK1	USA	Laboratory	Soil	SGRP^2^

^1 ^Centre International de Ressources Microbiennes, Thiverval-Grignon, France

^2 ^Saccharomyces Genome Resequencing Project, Sanger Institute, Cambridge, UK

^3 ^Washington University in St. Louis - School of Medicine, St. Louis, USA

### Culture Conditions and Measurements

For measuring accurately various life-history and metabolic traits, the strains were grown in the same artificial laboratory conditions. Growth kinetics were realized in two liquid media containing 3% Yeast Nitrogen Base with amino acids and differing by their glucose content, 1% and 15%. One percent glucose corresponds to a low concentration, commonly used in laboratory conditions, while 15% glucose is close to the concentration found in sap or fruits. We chose this nitrogen concentration to make sure that the glucose is the sole limiting nutrient. At 1% glucose concentration, yeast populations are supposed to grow first in fermentation then to switch to respiration. At 15% glucose concentration, yeast populations are supposed to grow only in fermentation, and are submitted to osmotic stress. The cultures were incubated at 30°C under 200 rpm agitation. In these conditions, yeast is exclusively reproducing by mitosis. After an overnight culture, around 10^6 ^cells were put into 40 mL fresh medium (30°C, 200 rpm). During growth kinetics, two samples (1 mL and 200 μL) were taken at seven time points (*t *= 4, 9, 13, 24, 48, 72 and 96 h) to estimate: (i) the glucose and ethanol concentrations, (ii) the population density and mean cell size measured with a counter Beckman Coulter^® ^Z2. Each growth kinetics was repeated independently from two to four times. Each replication was performed with a new colony from the reference stock.

### Life-history traits

**Figure 1:**
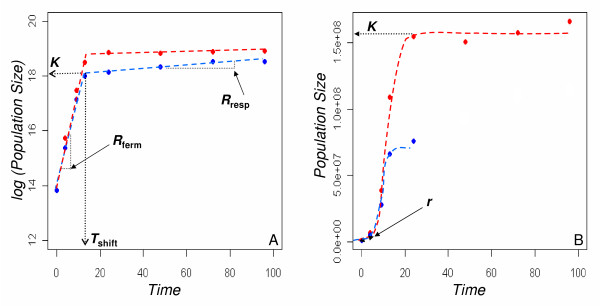
**Variation of population sizes overtime during the growth kinetics**. Examples of experimental data points (strain YPS1009) are presented with red (15% glucose) and blue dots (1% glucose). Two methods were used for fitting experimental data points and estimating life-history trait values. A) a segmented regression (red and blue broken lines) of log-transformed data points allowed us to estimate the reproduction rate in fermentation (*R*_ferm_), the time point of the diauxic shift (*T_shift_*), the population size at the end of the fermentation process (carrying capacity *K*), and the reproduction rate in respiration (*R*_resp_). B) an adjustment to a logistic model allowed us to estimate the intrinsic growth rate (*r*) and the carrying capacity (*K*).

Life-history traits were measured by analyzing the population dynamics. In batch culture, the changes over time of the cell number are classically analysed using a logistic model (exponential growth followed by a stationary phase) in order to estimate population dynamics parameters. However, a diauxic shift occurs in the 1% glucose medium dividing the dynamics into two parts: after a first period of growth in fermentation with production of ethanol, yeast shifts its metabolism and the respiration process takes place using ethanol as a carbon source. Therefore, the population dynamics was analyzed using two adjustment models (Figure 1).

First, the cell number over time was log-transformed, and a segmented regression model [19] was used to estimate the parameters of the transition between fermentation and respiration (*T*_shift_). The slope in fermentation gave the population reproduction rate in fermentation (*R*_ferm_), the population size at *T*_shift _gave the carrying capacity in fermentation and the slope after the *T*_shift _gave the population reproduction rate in respiration after the diauxic shift (*R*_resp_) (Figure 1A). This adjustment procedure was applied using the "segmented" package under R software. Although a diauxic shift is not supposed to occur in 15% glucose medium, some strains appear to have a small respiration phase in this condition (*R*_resp _different from zero) and we therefore used the method in the two media.

Second, a logistic model of population dynamics was fitted to experimental datapoints in fermentation (the points before *T*_shift_):

where *N*_*t *_is the population size at time *t, K *is the carrying capacity (maximum population size, reached when glucose is exhausted), *N*_0 _is the initial population size and *r *is the intrinsic reproduction rate (maximum rate of increase of the population, *i.e*. at the beginning of the kinetics when there is no competition). Fitting the growth kinetics with this model allowed estimating *K *and *r *(Fig 1B).

As expected, a significant positive correlation was observed between the intrinsic growth rate *r *estimated by the second procedure and *R*_ferm _obtained from the first procedure (*ρ*_15% _= 0.74 and *ρ*_1% _= 0.77). Therefore we chose to focus only on *R*_ferm_.

As ethanol was not completely consumed after 96 hours, when the experiments were stopped, we did not have access to the population size (carrying capacity), cell size and yield (population size × cell size) at the end of the respiration process. We thus focused on the cell size, carrying capacity and yield at the end of the fermentation process. Mean cell size (*S*_cell_) was therefore analyzed at the end of the fermentation process, *i.e*. at the time point the closest to *T*_shift_.

### Metabolic traits

#### *Glucose consumption rate and ethanol production*

Glucose and ethanol extracellular concentrations were measured using enzymatic kits (rBiopharm, Darmstadt, Germany). We estimated the glucose consumption rate as described in [18]. The glucose consumption rate at *T*_50 _(when 50% of the glucose was consumed) was divided by the population size at the same time point in order to infer a specific glucose consumption rate denoted *J*_spec _(g × min^-1 ^× cell^-1^). Note that the *T*_50 _falls during the exponential phase of the fermentation process in the two media (approximately after 8 hours and 20 hours respectively in the 1% and in the 15% glucose medium). We also measured the maximal quantity of ethanol produced and released in the medium (*Eth*_max_), which occurred at the end of the fermentation or at the beginning of the respiration process, *i. e*. close to *T*_shift_.

#### Fermentation yield

In microbiology, the yield is classically estimated through the measurement of the OD per unit of glucose consumed [20]. However, the correlation between OD value and biomass (product of the mean cell size by the population size [18]) is only *ρ *= 0.77. So we estimated directly the fermentation yield, *Y*_ferm_, using the ratio of the biomass over the quantity of glucose consumed. We did not consider the yield of respiration because we did not reach the plateau of this process.

### Microsatellite analysis

Yeast DNA extraction and microsatellite PCR amplifications were carried out as described in [21]. Eight microsatellites (three dinucleotide and five trinucleotide repeats) were analysed. They are located on 7 different chromosomes, mostly in non coding region.

### Statistical Analysis

A Principal Component Analysis was first conducted on all traits (both life-history and metabolic traits) averaged over replications to visualize the distribution of the strains on the plane defined by the two axes explaining the largest part of variation.

Then the factors accounting for the variation of each variable among media and strains were analyzed with a mixed analysis of variance model:

where *Z *is the quantitative variable (*K*, *S*_cell_, *R*_ferm_, *T*_shift_, *J*_spec_, *Eth*_max _or *Y*_ferm_), *Block *is the random block effect (experimental repetition) (*i *= 1, 2, 3, 4), *medium *is the medium effect (*j *= 1, 2), *ori *is the origin effect (*k *= 1, 2, 3, 4, 5), *Strain *is the random strain effect within habitat of origin (*l *= 1, 2, 3, 4, 5), Block*medium (random), medium*ori (fixed) and medium*Strain(ori) (random) are interaction effects and *ε *is the residual error.

For each trait, the normality and homogeneity of the residual distributions were studied. For all traits except *R*_resp _and *J*_spec_, a logarithmic transformation proved to be necessary to have normally and homogeneously distributed residues. The variables *R*_resp _and *J*_spec _displayed particular distributions. We chose to realize the ANOVA on the average values over the experimental repetitions for *R*_resp_, and on the log-transformed average values over the experimental repetitions for *J*_spec_. This resulted in homogeneity and normality of the residual distributions, but the ANOVA model used was less powerful:

where *Z *is the variable (*R*_resp _or *J*_spec_), *medium *is the medium effect (*i *= 1, 2), *ori *is the origin effect (*j *= 1, 2, 3, 4, 5), *Strain *is the random strain effect within industrial origin (*l *= 1, 2, 3, 4, 5), medium*ori is a fixed interaction effect and *ε *is the residual error.

The average performances of strains of different origins were compared using the least square estimation of *Y_k _*obtained by averaging the phenotypic value over strains from the same origin. Least square mean and variance estimates were obtained from JMP Procedure (SAS Institute Inc.). Significance of differences between means was assessed using Tukey HSD method.

### Genetic Correlations between life-history traits

The 19 strains were not phylogenetically independent. Based on microsatellite length differences, we computed a matrix of genetic Euclidian distances between all pairs of strains. This matrix was used to specify the variance structure in a multivariate generalized linear model. Correlations between traits were calculated based on variance/covariance component estimates by MCMC method. Prior distribution for variance and covariance components of the G-matrix (the genetic variance/covariance matrix) and of the R-matrix (the residual variance/covariance matrix) were chosen according to the package documentation (inverse Wishart distributions). These analyses were performed under R software using 'ape' [22] and 'MCMCglmm' (Hadfield, J: MCMC methods for Multi-response Generalised Linear Mixed Models: The MCMCglmm R package, submitted) packages.

As we tested for 20 correlations, we corrected *p*-values using a local FDR (False Discovery Rate) procedure [23].

### Relationships between life-history and metabolic traits

In each glucose condition, the variability of life-history traits was compared to the variability of metabolic traits, according to the regression model:

where *Y_il _*is the mean value over repetitions of a life-history trait of the strain *i *in the medium *l *and *X_il _*is the mean value over repetitions of a metabolic trait (*J*_spec_, *Eth*_max _or *Y*_ferm_) of the strain *i *in the medium *l*. *D *represents the phylogenetic distance matrix and *ε_il _*is the residual error term. We corrected *p*-values for multiple tests as previously.

## Results

We measured five life-history traits (the carrying capacity *K*, the mean cell size in fermentation *S*_cell_, the reproduction rate in fermentation *R*_fermv_, the reproduction rate in respiration *R*_resp _and the time to reach the carrying capacity in fermentation *T*_shift_) and three metabolic traits (the glucose consumption rate *J*_spec_, the maximal quantity of ethanol released in the medium *Eth*_max _and *Y*_ferm_) in a collection of 19 strains coming from various habitats (industry, forest, fruit, clinical, laboratory) and grown in two contrasted culture media (1% and 15% glucose).

### Effect of the glucose content of the medium

**Figure 2:**
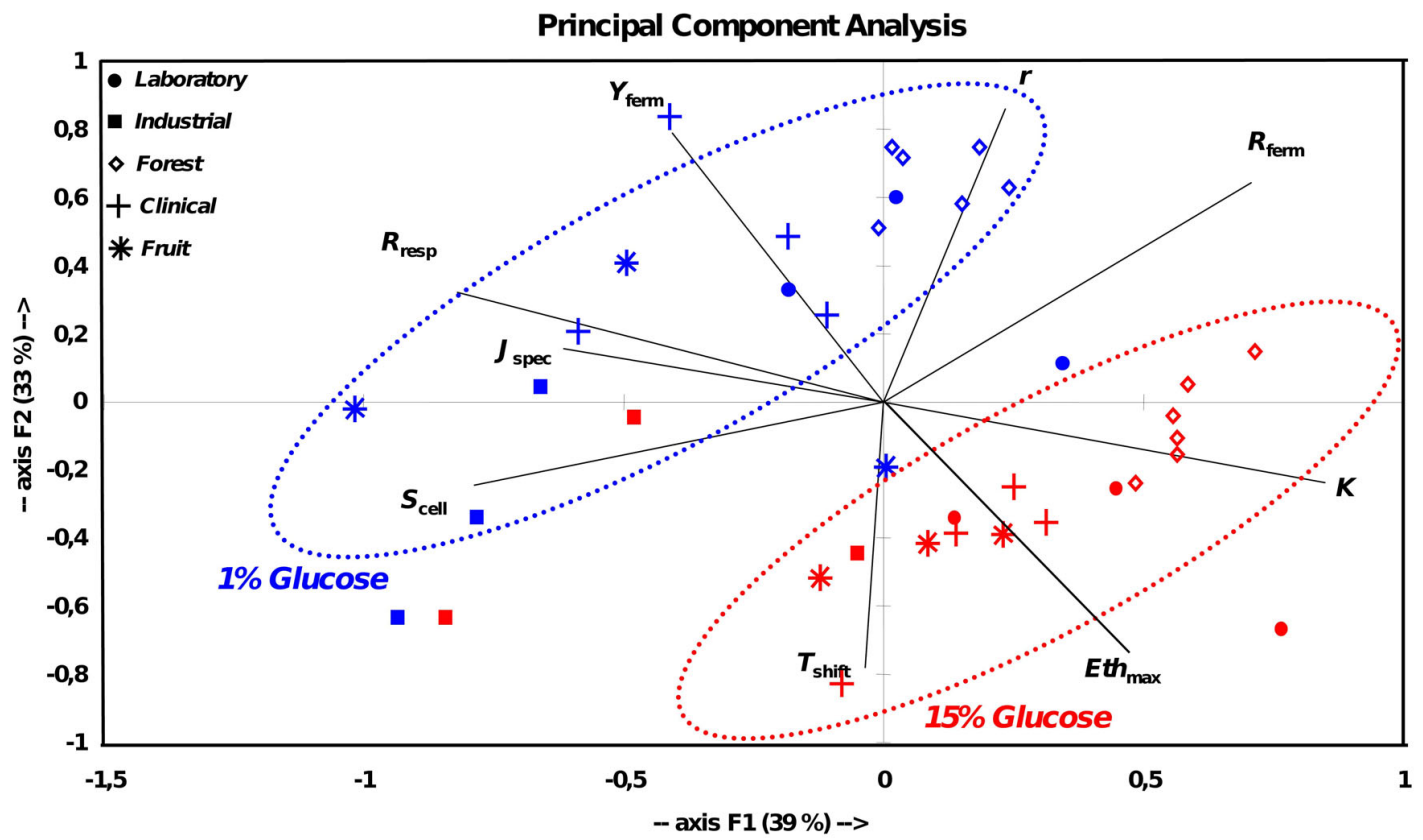
**Principal Component Analysis realized on the six life-history traits (*K, r, S*_cell_, *R*_ferm_, *R*_resp_, *T*_shift_) and on the three metabolic traits (*Y*_ferm_, *Eth*_max_, *J*_spec_)**. Filled circles correspond to laboratory strains, filled boxes to industrial ones, diamond to forest ones, crosses to clinical ones and stars to fruit ones. Red and blue symbols correspond to the two glucose conditions, respectively 15% and 1%.

We performed a Principal Component Analysis (PCA) to decompose the contribution of each quantitative trait on the variation between strains (Figure 2). The major part of the variation was explained by the first two axes (72%) of the PCA. A clear separation was observed between the strains grown in different glucose conditions. This separation was mainly explained by variation of metabolic traits (*J*_spec_, *Eth*_max _and *Y*_ferm_) and of two life-history traits: *K*, which should be the direct reflect of the resource availability in the medium, and *R*_resp_, because respiration is present only in the 1% glucose medium. The ANOVA confirmed that the medium effect is very large for these traits (Tables 2, 3). The variables *Eth*_max_, *K *and *T*_shift _increased with glucose content in the medium, while *Y*_ferm_, *J*_spec _and *R*_resp _had opposite behaviors. *R*_resp _was nearly equal to 0 in the 15% glucose medium. Surprisingly, in average, *S*_cell _and *R*_ferm _were not significantly affected by the glucose content of the medium.

**Table 2:** Analysis of variance for five life-history traits: the carrying capacity of fermentation (*K*), the mean cell size (*S*_cell_), the reproduction rate in fermentation (*R*_ferm_), the reproduction rate in respiration (*R*_resp_) and the time to reach the carrying capacity in fermentation (*T*_shift_).

		*K*		*S*_cell_		*R*_ferm_		*T*_shift_		*R*_resp_		
***Sources of Variation***	***df***	***MS***	***F***	***MS***	***F***	***MS***	***F***	***MS***	***F***	***df***	***MS***	***F***

*Block*	3	0.17	0.85	7 × 10^-3^	91	0.07	25	0.03	2.50			
*medium*	1	22.05	42.85**	3.42 × 10^-3^	0.29	0.13	2.77	0.35	8.74*	1	3 × 10^-4^	79.19**
*ori*	4	8.72	8.11**	0.27	12.79**	1.71	5.35**	0.19	2.11	4	1.28 × 10^-5^	0.99
*Strain(ori)*	14	1.16	2.81*	0.02	1.76	0.33	8.01**	0.10	2.55*	14	1.33 × 10^-5^	3.46*
Block*medium	3	0.20	8.43**	5.8 × 10^-4^	0.75	0.02	1.86	0.01	1.83			
medium*ori	4	0.46	1.19	5.15 × 10^-3^	0.43	0.21	5.34**	0.12	3.35*	4	6.63 × 10^-6^	1.72
medium*Strain(ori)	14	0.41	17.45**	0.01	15.96**	0.04	4.87**	0.04	6.73**			

*df *: degrees of freedom, *MS *: Mean Square, *F *: Fisher's *F*

* *p *< 0.05, ** *p *< 0.01

Block and Strain are main random effects while medium (1 and 15% glucose) and habitat of origin (*ori*: forest, industrial, clinical, fruit and laboratory) are main fixed effects.

**Table 3:** Analysis of variance for three metabolic traits: the yield of the fermentation process (*Y*_ferm_), the maximal quantity of ethanol produced in the medium (*Eth*_max_), and the specific glucose consumption rate when 50% of the glucose was consumed (*J*_spec_).

		*Y*_ferm_		*Eth*_max_		*J*_spec_		
***Sources of Variation***	***df***	***MS***	***F***	***MS***	***F***	***df***	***MS***	***F***

*Block*	3	0.23	0,89	0.93	19.86*			
*medium*	1	75.41	148,68**	179.63	2436.56**	1	4.59	12.21**
*ori*	4	5.11	5,20**	0.10	2.27	4	0.15	0.25
*Strain(ori)*	14	1.06	3,02*	0.05	0.91	14	0.59	1.57
Block*medium	3	0.26	9,86**	0.05	3.13*			
medium*ori	4	0.84	2,57	0.09	1.87	4	0.37	0.98
medium*Strain(ori)	14	0.35	13,28**	0.05	3.45**			

*df *: degree of freedom, *MS *: Mean Square, *F *: Fisher's *F*

*p < 0.05

**p < 0.01

Block and Strain are main random effects while medium (1 and 15% glucose) and habitat of origin (*ori*: forest, industrial, clinical, fruit and laboratory) are main fixed effects.

### Genetic variation of life-history and metabolic traits

Within each glucose condition, the strains appeared to be clustered according to their habitat of origin, and the variables that contributed mainly to this structure were life-history traits (*r, S*_cell_, *R*_ferm _and *T*_shift_). The "habitat of origin" had a significant effect on *K, S*_cell_, *R*_ferm _and *Y*_ferm _(Table 2 &3). Globally, industrial strains had a large cell size, reproduced slowly, reached a low carrying capacity and produced a weak biomass per unit of resource consumed (Table 4). On the opposite, laboratory and forest strains had a small cell size, reproduced quickly, reached a high carrying capacity and were very efficient in converting glucose into biomass (Table 4). Clinical and fruit strains were intermediate between these two contrasted groups, fruit strains being closer to industrial strains and clinical ones to forest and laboratory ones. Significant genetic variation between strains coming from a same habitat of origin was also found (*Strain(ori) *effect) for all traits but for *S*_cell _(Table 2 &3). Note that genetic main effects (*ori *and *Strain(ori)*) were significant neither for *Eth*_max _nor for *J*_spec_.

**Table 4:** Main effect of the habitat of origin on life-history and metabolic traits variation

	Industry	Fruit	Clinical	Laboratory	Forest
***K***	2.12 10^7 B^	4.36 10^7 AB^	4.96 10^7 AB^	9.99 10^7 A^	9.80 10^7 A^
***S*_cell_**	6.36^A^	5.21^BC^	5.36^B^	4.81^BC^	4.80^C^
***R*_ferm_**	0.18^B^	0.23^AB^	0.26^AB^	0.31^AB^	0.35^A^
***Y*_ferm_**	3.80 10^6 B^	6.26 10^6 AB^	7.57 10^6 AB^	1.26 10^7 A^	1.22 10^7 A^

The average value of the reproduction rate in fermentation (*R_ferm_*), the carrying capacity (*K *in number of cells per mL), the cell size (*S*_cell _in μm), and the yield (*Y_ferm _*= *K *× *S*_cell_) at the end of the fermentation process is reported for each habitat of origin. Significance of differences was assessed by HSD Tukey tests. Levels that are not connected by the same letter are significantly different.

### Genotype-by-environment interactions

The medium*Strain(ori) interaction effect was significant for all traits. The medium*origin interaction effect was only significant *R*_ferm _and *T*_shift_. Industrial populations displayed a significantly higher *R*_ferm _value in 15% glucose than in 1% glucose (*p *< 0.05), while the other types of population harbour similar *R*_ferm _values in the two media. Industrial populations displayed a lower *T*_shift _value in 15% glucose than in 1% glucose, while it was the opposite for the other types of populations.

### Genetic correlations between life-history traits

Correlations between life-history traits were studied using the average trait value for each strain in each glucose condition and taking into account the genetic relationships between strains (Table 5).

**Table 5:** Pearson correlation coefficients between life history traits

Life-History Traits	*K*	*S*_cell_	*R*_ferm_	*R*_resp_	*T*_shift_
***K***	\	-0.79^b ^[-0.94, -0.51]	0.87^b ^[0.54, 0.94]	-0.63^b ^[-0.85, -0.18]	-0.04^NS ^[-0.49, 0.46]
***S*_cell_**	-0.73^b ^[-0.88, -0.38]	\	-0.72^b ^[-0.91, -0.37]	0.82^b ^[0.49, 0.93]	-0.23^NS ^[-0.65, 0.27]
***R*_ferm_**	0.87^b ^[0.65, 0.95]	-0.71^b ^[-0.89, -0.37]	\	-0.54^a ^[-0.82, -0.01]	-0.37^NS ^[-0.72, 0.15]
***R*_resp_**	-0.79^b ^[-0.94, -0.23]	0.73^a ^[0.17, 0.92]	-0.73^a ^[-0.93, -0.11]	\	-0.46^NS ^[-0.78, 0.03]
***T*_shift_**	-0.74^b ^[-0.91, -0.40]	0.39^NS ^[-0.09, 0.72]	-0.81^b ^[-0.93, -0.52]	0.46^NS ^[-0.17, 0.86]	\

Over the diagonal: 15% glucose medium; below the diagonal: 1% glucose medium.

^a ^Pearson correlation coefficients becoming non-significant after the correction for multiple tests (see Material & Methods)

^b^Pearson correlation coefficients still significant after the local FDR procedure

*NS*: Non Significant

95% confidence interval of the estimation of the correlation coefficients are given between brackets

**Figure 3:**
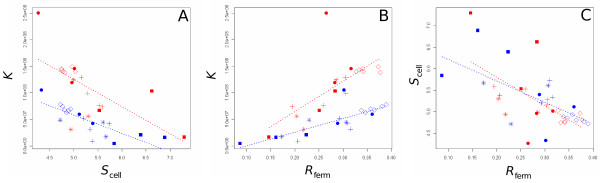
**Genetic correlations between life-history traits**. Each point corresponds to the mean value of traits for a strain in a glucose condition. Pearson correlation coefficients were calculated in each glucose condition between (A) *K *and *S*_cell_, (B) *K *and *R*_ferm_, (C) *S*_cell _and *R*_ferm_. Same symbols as in Figure 2.

We detected a significant negative correlation between the carrying capacity *K *and the *S*_cell _in each glucose condition (*ρ*_15% _= - 0.79 and *ρ*_1% _= - 0.73) (Figure 3A). A significant positive correlation was observed between the carrying capacity and the *R*_ferm _in each glucose condition (*ρ*_15% _= 0.87 and *ρ*_1% _= 0.87) (Figure 3B). Finally, a significant negative correlation between *S*_cell _and *R*_ferm _in each glucose condition (*ρ*_15% _= - 0.63 and *ρ*_1% _= - 0.79) was found in agreement with the two previous relationships (Figure 3C).

The time to reach the carrying capacity (*T*_shift_) was negatively correlated with *R*_ferm _in the 1% glucose medium (*ρ*_1% _= - 0.81), indicating that, the faster cells reproduced, the earlier they reached the carrying capacity in fermentation. More interestingly, a significant positive correlation between *S*_cell _and *R*_resp _(*ρ*_15% _= 0.82 and *ρ*_1% _= 0.73) was detected.

Consequently, whatever the quantity of resource, life-history trait variation in *S. cerevisiae *populations distributes between two extreme life-history strategies: populations of small cells reproduce quickly in fermentation and reach rapidly a high carrying capacity at the expense of a small reproduction rate in respiration, and populations of large cells reproduce slowly in fermentation, reach a lower carrying capacity but reproduce faster in respiration.

### *Strategies of resource utilization*

**Figure 4:**
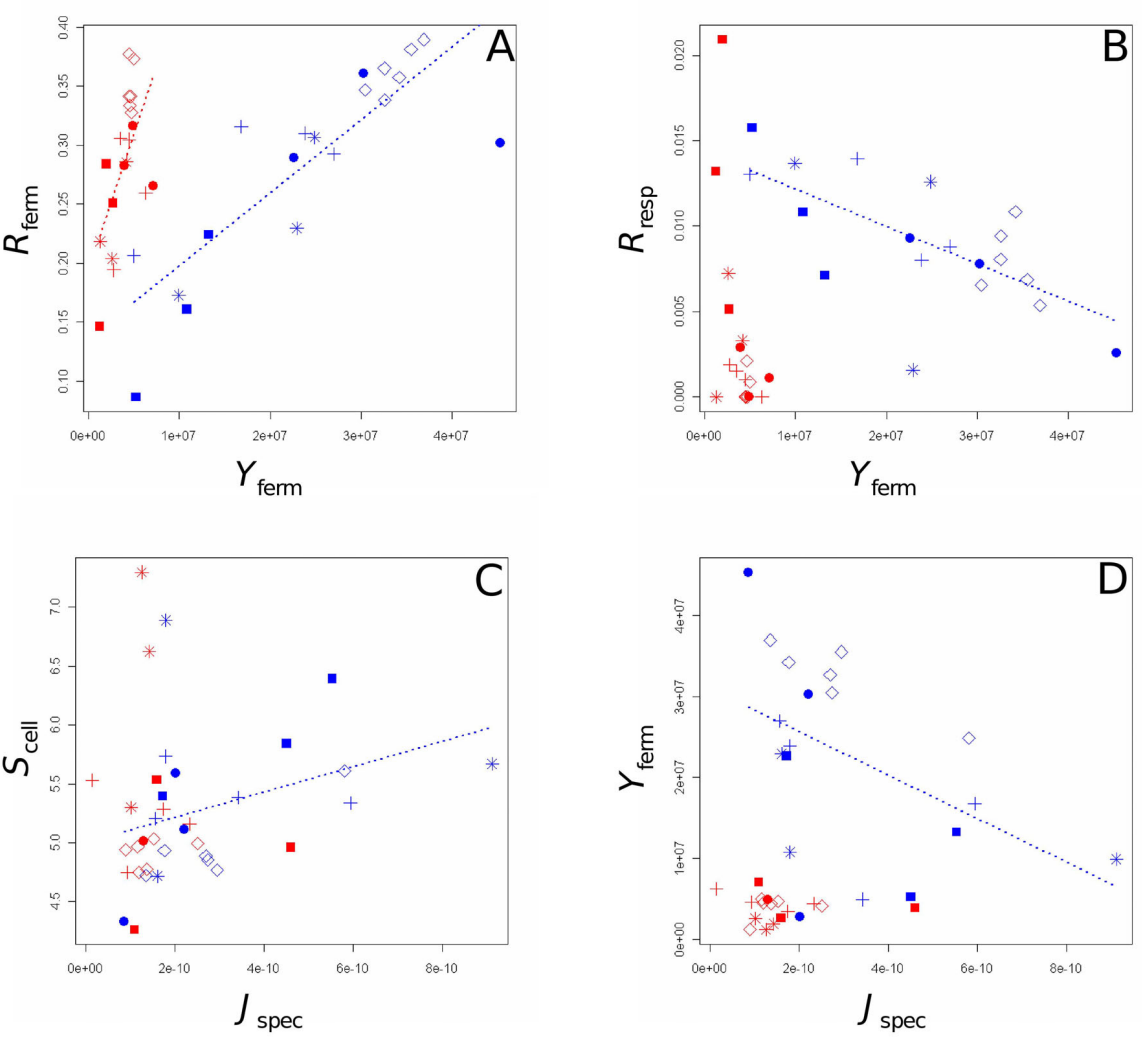
**Relationships between life-history traits and metabolic traits**. Each point corresponds to the mean value of traits for a strain in a glucose condition. Regressions were calculated in each glucose condition between (A) *R*_ferm _and *Y*_ferm_, (B) *R*_resp _and *Y*_ferm_, (C) *S*_cell _and *J*_spec _and (D) *Y*_ferm _and *J*_spec_. Same symbols as in Figure 2.

Relationships between each life-history and metabolic traits were studied by linear regression using, as we did for correlations, the average trait value for each strain in each glucose condition and taking into account the genetics relationships between strains. The *R*_ferm _was positively correlated with *Y*_ferm _in the two glucose conditions (*R^2^*_15% _= 80%, *p *< 10^-4 ^and *R^2^*_1% _= 84%, *p *< 10^-4^) (Figure 4A), while *R*_resp _was negatively correlated to *Y*_ferm _in 1% glucose (*R^2^*_1% _= 68%, *p *= 0.003) (Figure 4B). These opposite behaviours gave more weight to the marginally significant trade-offs observed in both environments between the two different reproduction rates (Table 5). Strains that were very efficient for the fermentation process (high reproduction rate and high yield in biomass) seemed to be less efficient in respiration.

In both media, *S*_cell _correlated negatively with *Eth*_max _(*R^2^*_15% _= 68%, *p *< 0.001 and *R^2^*_1% _= 67%, *p *< 0.024) (not shown). In the 15% glucose medium, where fermentation is the main process, an increase of the *R*_ferm _and of *K *were significantly correlated with *Eth*_max _(*R^2^*_15% _= 47%, *p *< 0.013 and *R^2^*_15% _= 61%, *p *= 0.001), while in 1% glucose, where respiro-fermentation occurs, the *R*_resp _was significantly negatively correlated to *Eth*_max _(*R^2^*_1% _= 26%, *p *< 0.0265). In other words, strains with small cell size that were efficient in the fermentation process released high amount of ethanol, while strains of large cell size, less efficient in fermentation, produced less ethanol and/or were more able to re-consume it as a carbon source during the respiration process.

Finally, in the 1% glucose medium, *J*_spec _was negatively correlated to the carrying capacity *K *and to the *Y*_ferm _(*R^2^*_1% _= 61%, *p *< 0.04 and *R^2^*_1% _= 58%, *p *= 0.032, Figure 4D) and positively to the *S*_cell _(*R^2^*_1% _= 49%, *p *= 0.05) (Figure 4C), while in the 15% glucose medium no correlation between *J*_spec _and any life-history trait was observed.

## Discussion and Conclusions

### Life-history strategies in wild and domesticated populations of S. cerevisiae

In a previous study, two opposite genetically-based life-history strategies have been described from the study of a collection of twelve industrial strains of *S. cerevisiae*. Some strains (the ''grasshoppers'') consumed quickly glucose, reached a big cell size at the expense of their carrying capacity, whereas other strains (the ''ants'') consumed slowly glucose, reached a smaller cell size, but had a higher carrying capacity [18]. We extended the panel of *S. cerevisiae *strains to non-industrial populations to determine whether these two life-history strategies had arisen as a consequence of domestication for food processing utilization or could be found in natural habitats. We showed that these two life-history strategies can be found in other habitats and that the range of trait variation between the two opposite strategies is larger when adding populations from non-industrial sources. In addition, the definition of the two opposite life-history strategies have been refined by analysing not only fermentation but also the respiration process: the "ants" reproduce quickly in fermentation, reach a large carrying capacity but have a small cell size and have a low reproduction rate in the respiration process, whereas the "grasshoppers" reproduce slowly in fermentation, reach a small carrying capacity but cells are big and their reproduction rate in respiration is high. Moreover, the "grasshoppers" have higher or equal glucose consumption rate than "ants" while the maximum quantity of ethanol released in fermentation is lower. This suggests that "grasshoppers" store resources inside the cell rather than secrete secondary products to cross-feed or poison competitors during fermentation. Their performance during the respiration process on the other hand might be related to a better ethanol consumption rate as well as reallocation of intra-cellular stock. The distribution of glycolysis carbons into the different intra and extra cellular metabolites need to be studied for getting more insight into the physiological basis of "ant" and "grasshopper" life-history strategies.

### Niche-driven evolution of S. cerevisiae phenotypes

A significant "habitat of origin" effect for most of the life-history traits (*K, S*_cell _and *R*_ferm_) and for *Y*_ferm _was found. Despite a broad geographical origin, strains of the same habitat had similar life-history strategy. Compared to the other strains, the industrial strains can all be considered as "grasshoppers", even though the distinction between "ant" and "grasshopper" life-history strategies has been initially defined within this group. The laboratory and forest strains can be considered as extreme "ants" and the clinical and fruit strains as intermediate between these two groups (Table 4). Phenotypic homogeneity of strains coming from a same ecological niche supports the idea of genetic exchange among strains coming from similar habitats, as highlighted by phylogenomics studies for wine, laboratory and clinical-fruit strains [12-14,16,17,24]. In addition, homogenizing selection may lead to phenotypic convergence in geographical distant but similar habitats. This hypothesis seems to explain why the three sequenced laboratory strains (S288C, SK1 and Y55), which are the most genetically distant strains [16], have close life-history traits. In the case of forest strains our data also confirm a recent study on the variation of stress responses showing that oak strains are phenotypically more similar than expected from their genetic relationships [25]. Altogether, these results support the idea of a niche-driven evolution of *S. cerevisiae *with phenotypic convergence of populations living in similar habitat.

### The harshness of the winter season may be one of the key factors of the differentiation between "ants" and "grasshoppers"

The close phenotypic proximity between laboratory strains and forest strains, both having "ant" life-history strategies, is striking. Thinking about the environmental conditions common to these two groups of strains may help understanding what drives the evolution towards "ant" or "grasshopper" strategies. Both oak and laboratory strains appeared to live in low resource media. Oak strains have been isolated from bark or soil and laboratory strains are often grown in 1 or 2% glucose media. A lack of resource may lead to an "ant" strategy. Another explanation can be that both oak and laboratory strains have undergone freeze-thaw cycles related to environmental seasonality in forest or experimental utilization in research. Oak related strains have been demonstrated to be freeze-thaw resistant which have been hypothesized as an important advantage to survive to winter niche [25]. On the opposite, "grasshopper" strains would not have had the opportunity to adapt to multiple freeze-thaw cycles because of their habitat of origin. The fruit strains used are coming from Philippines, Hawaii and Bahamas, and have probably never experienced freezing before their isolation. Similarly, clinical isolates are expected to live at human body temperature even though their exact mode of transmission and life cycle are unknown. Finally, industrial strains are probably living in quite controlled environments with no drastic temperature changes as expected in wine cellars [26].

This hypothesis is supported by our knowledge on freeze tolerance which is expected to be higher in smaller cells. Freezing tolerance has been related to cell size in *S. cerevisiae *[27,28], *Torulaspora delbrueckii *[29], *Candida utilis, Escherichia coli, Lactobacillus plantarum*, and the human leukemia K562 cells [30] and may have driven the evolution towards "ant" life-history strategy. A physical explanation could be that the larger surface-to-volume ratio of the small cells makes easier the release of water out of the cell and the decrease of intracellular pressure. Moreover, the formation of ice crystals leading to the mechanical disruption of cell components may be higher in large cells [30]. Selection experiments under variable "winter-season" length and temperature should be conducted to test these hypotheses.

### Tragedy of the commons in S. cerevisiae populations - Where are we?

Competition for resources can lead to a "tragedy of the commons", *i. e*. a dilemma between using resource selfishly to the detriment of the long-term survival of the population, or saving resource for the whole community at the expense of its own reproduction. These two opposite types of resource utilization have already been described in yeast and are often illustrated by the trade-off between the rate of glucose uptake (supposed to be high in selfish strains) and yield (supposed to be improved in cooperative strains) [20]. However, the trade-off between the yield and the resource uptake rate is commonly found in low glucose media but does not seem to be detected in richer media. In 15% glucose medium, where fermentation is the main physiological process, the trade-off between the yield and the resource uptake rate was not detected among industrial strains [18] and was not detected here in a panel of strains representing a larger genetic diversity. This gives evidence for an environment-dependent rate/yield trade-off, which reflects the biochemical trade-off between fermentation and respiration, but it does not seem to occur in fermentation.

Competition for resources and social conflict between "ants" and "grasshoppers" does not seem to commonly occur since the two opposite life-history strategies appear to be maintained in distinct ecological niches. Intermediary phenotypes, such as clinical and fruit strains, which have been previously described as resulting from exchanges between isolated genetic groups because of the mosaic composition of their genomes [16,17], could be the evolutionary outcomes of the competition and/or hybridization between "ants" and "grasshoppers". Human body and fruit have indeed been proposed to be environments that are regularly colonized by yeast coming from various geographical and ecological origins [16,17] and thus could be locations where strains of the two opposite life-history strategies meet and compete. However, the outcome of their competition remains difficult to predict. In a one-season time-scale in fermentation, "ants" may win the competition: they save resources, reproduce fast and invest preferentially resources in the carrying capacity at the expense of their own cell size. In a context of long-term competition through multiple seasons with fermentation/respiration cycles, "grasshoppers" should be the "winners". Although they reproduce slowly and reach a lower carrying capacity, they use resources quickly and store them inside the cell which may allow a better survival to starving conditions. In fermentation, they produce a small amount of "toxic" ethanol. In respiration, they may re-consume efficiently ethanol as a carbon source since they reproduce quickly. "Ants" and "grasshoppers" could both be considered as "selfish competitors" depending on the moment of the season.

Therefore, determining the cooperative or selfish nature of each of the two life-history strategies raised no consensus and depends both on the environment and on the time scale. Mathematical modelling and experimental validations of short and long-term competition between "ants" and "grasshoppers" in various habitats should be developed to study the evolutionary outcome of competitions between them.

## Authors' contributions

AS & DS conceived and designed the experiments. AS, TN, JS & AB performed the experiments. AS, TN, JS & DS analyzed the results. AS, DdV & DS wrote the paper. All authors read and approved the final manuscript.
